# Cost-effectiveness analysis of three surgical approaches for parathyroidectomy in secondary hyperparathyroidism patients

**DOI:** 10.3389/fendo.2024.1495818

**Published:** 2025-01-27

**Authors:** Qila Sa, Yinghui Zhou, Guangming Cheng, Boyuan Nan, Yingnan Feng, Ningyuan Zhang, Wantao Xie, Wei Zhang

**Affiliations:** ^1^ Department of Hepatobiliary Pancreatic Splenic and Thyroid Surgery, General Hospital of Northern Theater Command, Shenyang, China; ^2^ Dalian Medical University, General Hospital of Northern Theater Command Training Base for Graduate, Shenyang, China; ^3^ Chinese Medical University, General Hospital of Northern Theater Command Training Base for Graduate, Shenyang, China

**Keywords:** secondary hyperparathyroidism, parathyroidectomy, cost-effectiveness analysis, Markov model, surgical approaches

## Abstract

**Background:**

There are three main surgical treatment options for secondary hyperparathyroidism (SHPT): subtotal parathyroidectomy (sPTX), total parathyroidectomy with auto-transplantation (tPTX+AT), and total parathyroidectomy (tPTX). However, a debate regarding which of these surgical methods is optimal has been ongoing. Aim of this study is to compare medical costs and final outcomes associated with the three surgical approaches for the entire treatment duration, aiming to identify the most cost-effective surgical method.

**Methods:**

Based on previous research data from domestic and international studies, as well as data from on-site surveys, TreeAge Pro 2022 software was used to construct a Markov model for the surgical treatment of SHPT patients. The model was run using data from the 2022 registered population of end-stage renal disease dialysis patients in China (1 million) as baseline cohort. Main indicators for this analysis are total cost, quality-adjusted life years, and incremental cost-effectiveness ratio (ICER). The study period is 10 years post-surgery, with a discount rate of 5% per year. Uncertainty in the model was assessed using one-way sensitivity analysis and probabilistic sensitivity analysis (PSA).

**Results:**

The costs incurred by SHPT patients undergoing sPTX, tPTX, and tPTX+AT within 10 years post-surgery are $7042.54, $9983.00, and $11435.60, respectively, with total utilities generated being 13.23 QALYs, 18.76 QALYs, and 18.69 QALYs. Compared to sPTX, the incremental costs and incremental effects of tPTX and tPTX+AT are $2,924.71 and $4,456.66, with 5.53 QALYs and 5.46 QALYs, respectively. The ICER for tPTX and tPTX+AT groups are $532.13/QALY and $805.10/QALY, respectively, which are well below our set willingness-to-pay (WTP) threshold. Sensitivity analysis results indicate that varying any parameter within a certain range over the given time interval will not cause the ICER to exceed the WTP threshold and will not reverse the primary analysis results.

**Conclusion:**

In the Chinese healthcare system, tPTX is considered the most cost-effective treatment for refractory hyperparathyroidism, when compared to tPTX+AT and sPTX.

## 1 Introduction

Secondary hyperparathyroidism (SHPT) is caused by hypocalcemia and hyperphosphatemia due to the disorder of the endocrine metabolic environment in patients with end-stage renal disease, resulting in hyperparathyroidism, parathyroid hyperplasia, and a series of clinical symptoms (1, 2). Clinically, it is characterized by a continuous rise in parathyroid hormone levels, enlargement of the parathyroid glands, and persistent bone pain, itching, insomnia, restless leg syndrome, and pathological fractures. This condition affects patient’s quality of life and can even lead to an increased risk of death (3, 4).

Currently, primary clinical treatments for SHPT include surgical and medical approaches. Medical treatment primarily involves use of calcimimetics. Researches have shown that calcimimetics, such as cinacalcet, can effectively reduce serum PTH levels and diminish the volume of parathyroid gland (5–7). However, patients with refractory SHPT, those for whom medical treatment is ineffective, or those who are intolerant to medication still require surgical intervention (8, 9). Currently, primary surgical treatments include total parathyroidectomy (tPTX), total parathyroidectomy with auto-transplantation (tPTX+AT), and subtotal parathyroidectomy (sPTX). However, a debate regarding the optimal surgical approach among these three methods remains ongoing. Several scholars have published clinical studies, systematic reviews, and meta-analyses to offer recommendations to surgeons. However, to date, there are no definitive guidelines identifying the optimal surgical method.

Our aim in this study was to determine which surgical method is most cost-effective and to offer recommendations for surgeons in selecting the appropriate treatment for SHPT.

## 2 Materials and methods

### 2.1 Model design

This study utilized the 2022 Chinese registry of end-stage renal disease dialysis patients (1 million) as the baseline population, assuming all these patients had SHPT and were awaiting surgical treatment. Then we used TreeAge Pro 2022 (Williamstown, MA) to develop a Markov model for the surgical treatment of SHPT from a societal perspective. The model had an annual cycle duration and terminated 10 years post-surgery. The model’s health states included post-surgical SHPT cure, surgery-induced death, post-surgical hypocalcemia, and other serious complications such as recurrent laryngeal nerve injury, bleeding, cardiovascular events, and infections.

### 2.2 Management strategy

**Figure 1:**
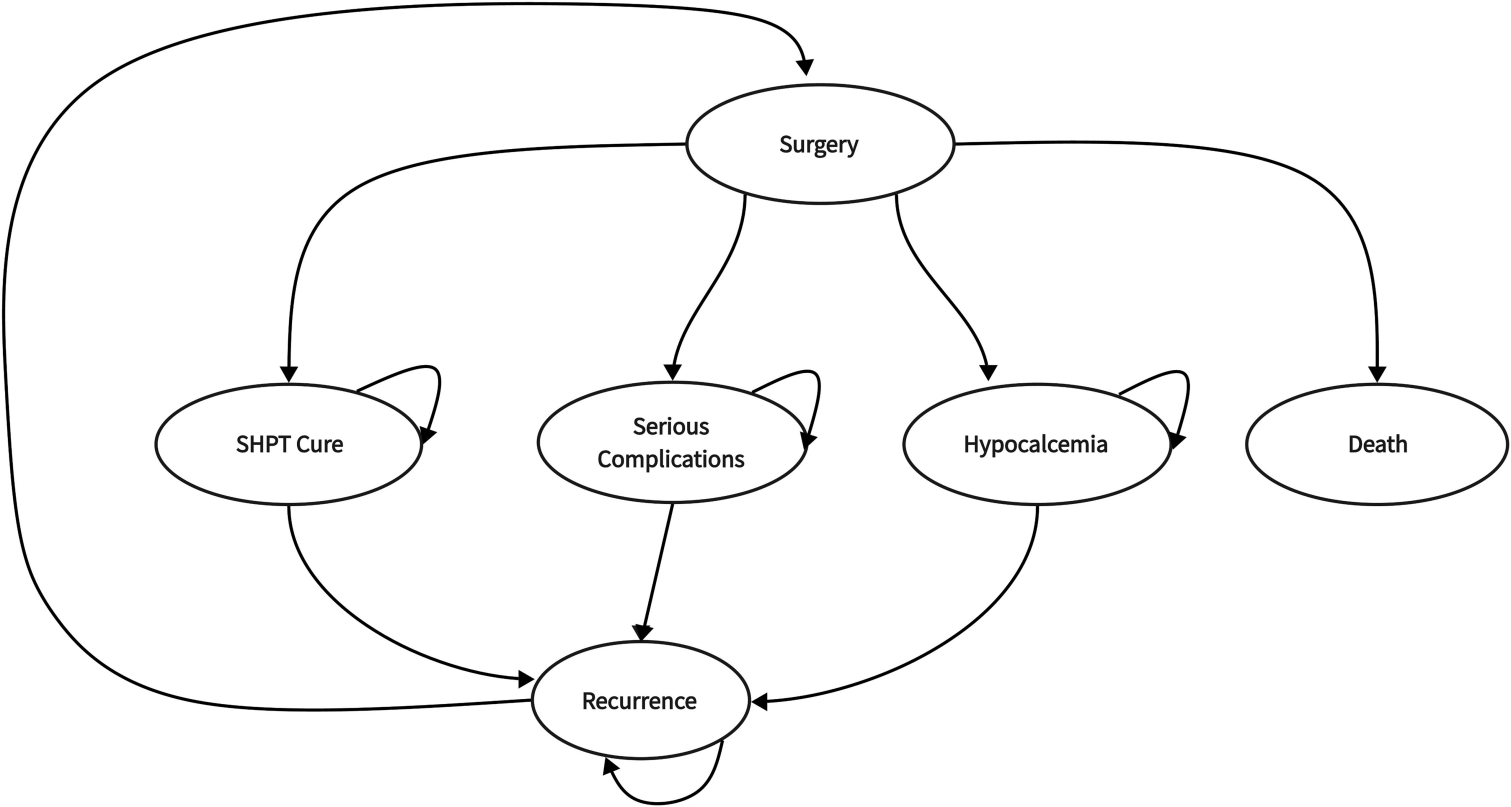
Simplified model of treatment strategies with health states.

In this research, the surgical strategies for SHPT included tPTX, sPTX, and tPTX+AT. In tPTX+AT, the grafts were transplanted into patient’s right forearm. After surgical treatment, patients transitioned into four predefined health states: post-surgical SHPT cure, surgery-induced death, post-surgical hypocalcemia, or other serious complications. A cohort of patients experiencing postoperative hypocalcemia received calcium supplementation therapy through administration of oral calcium carbonate tablets and calcitriol tablets. With the exception of state of death, the other three states all fall into two states of SHPT being controlled or recurrence. In tPTX+AT group, patients may experience two types of recurrence: at the right forearm graft site or in the neck. All patients with recurrence underwent reoperation, thus completing a cycle (**Figure 1**).

### 2.3 Data sources

Main parameter values of the Markov model for cost-effectiveness of surgical treatment in patients with SHPT are presented in **Table 1**. Our study’s data inclusion criteria: original research or meta-analyses published in domestic and international journals from 2015 to 2023, focusing on parathyroidectomy alone without transcervical thymectomy. The surgery must have involved the complete removal of four parathyroid glands, with no definitive ectopic parathyroid glands identified in preoperative examinations or intraoperative exploration. Exclusion criteria: presence of ectopic parathyroid glands, failure to completely remove all four parathyroid glands during surgery, and data indicating residual parathyroid glands identified after tPTX+AT or tPTX procedures.

**Table 1:** Base case values and ranges used in sensitivity analysis.

Name	Root Definition	Range used for sensitivity analysis		Distribution	References
		Low	High		
Discount rate	0.05	0	0.06	Beta	(10)
Risk of complications after sPTX	0.15	0.12	0.18	Beta	(11)
Risk of complications after tPTX	0.089	0.0712	0.09	Beta	(12)
Risk of complications after tPTX+AT	0.089	0.0712	0.1068	Beta	(12)
Mortality of sPTX	0.012	0.0096	0.0144	Beta	(11)
Mortality of tPTX	0.008	0.0064	0.0096	Beta	(11)
Mortality of tPTX+AT	0.008	0.0064	0.0096	Beta	(11)
Risk of hypocalcemia after sPTX	0.49	0.392	0.588	Beta	(13)
Risk of hypocalcemia after tPTX	0.90	0.72	0.902	Beta	(14)
Risk of hypocalcemia after tPTX+AT	0.83	0.664	0.9	Beta	(14)
Risk of recurrence after tPTX	0.015	0.012	0.018	Beta	(15)
Risk of recurrence after sPTX	0.084	0.0672	0.1008	Beta	(16)
Risk of recurrence after tPTX+AT	0.083	0.0664	0.0996	Beta	(16)
Utility of death	0	–	–	Beta	–
Utility of hypocalcemia	0.894	0.794	0.994	Beta	(17)
Utility of complication	0.89	0.79	0.99	Beta	(18)
Utility of SHPT controlled	1	–	–	Beta	–

#### 2.3.1 Epidemiology

In the model, epidemiological parameters used, including rates of post-surgical hypocalcemia, rates of other serious complications, mortality rates, recurrence rates, and rates of post-surgical SHPT cure, were all obtained from previously published literature, both domestic and international.

Since the previously published domestic and international literature did not directly provide mortality rates for the three types of surgery, we assumed that the 30-day postoperative mortality rates represent surgery-related mortality rates. Previous studies have shown that there is no significant difference in surgery time between tPTX and tPTX+AT (14). Therefore, we assumed that the mortality rates and the rates of other serious complications for these two surgical methods are same.

#### 2.3.2 Cost

The costs included in this study primarily consist of direct medical costs, which encompass medication fees, examination fees, laboratory fees, surgical operation and material costs, nursing fees, and hospitalization costs. These were obtained from on-site surveys conducted in representative tertiary hospitals in China. The total of 207 cases of SHPT surgeries performed at this hospital, including 130 cases of tPTX, 23 cases of tPTX+AT, 47 cases of sPTX, 5 cases of SHPT recurrence surgeries, and 2 cases of the right forearm graft removal surgeries. As several datasets followed a normal distribution, the mean was used to construct the model (**Table 2**). We also used 5% discount rate to eliminate the effects of inflation.

**Table 2:** Base case values of cost.

Name	Sample Size	Minimum Value	Maximum Value	Average	95% Confidence Interval		Standard Deviation
					Min	Max	
Cost of re-PTX after sPTX and tPTX ($)	5	2177.08	3836.25	3091.88	2281.57	3902.19	652.60
Cost of re-PTX after tPTX+AT ($)	2	1365.38	1974.62	1670.00	–	–	–
Cost of sPTX ($)	23	2384.29	3876.65	3032.41	2830.11	3234.71	467.83
Cost of tPTX ($)	130	1460.86	3967.39	3030.53	2951.85	3109.21	455.20
Cost of tPTX+AT ($)	47	2333.93	4753.96	3527.53	3356.20	3698.86	583.52

#### 2.3.3 Utility

Using quality-adjusted life years (QALYs) to assess health outcomes, ranging from 0 to 1. Post-surgical SHPT cure equals 1, death equals 0, the utility values of post-surgical hypocalcemia and other serious complications were obtained from published literature both domestically and internationally.

### 2.4 Base-case analysis

Incremental Cost-Effectiveness Ratio (ICER) is employed to evaluate SHPT surgical strategies. ICER refers to the cost required per additional QALY. ICER is computed using TreeAge Pro 2022 software with formula: ICER = (Cost of Strategy 2 - Cost of Strategy 1)/(QALY of Strategy 2 - QALY of Strategy 1). QALY values are determined by multiplying the survival time in a specific health state by the health utility of that state. The willingness to pay (WTP) threshold is set at $12,700 USD, based on China’s per capita Gross Domestic Product (GDP) in 2023.

### 2.5 Sensitivity analysis

We conducted one-way sensitivity analysis to assess the stability of the Markov model. By varying a single parameter within its range while keeping other parameters constant, we calculated changes in ICER to generate tornado diagrams. Probability sensitivity analysis was employed to evaluate cost-effectiveness under the WTP threshold, assigning specific distributions to model parameters. Monte Carlo simulations were conducted with 1,000 iterations and recalculations on a cohort of 1,000 patients to further elucidate the optimal treatment strategy under conditions of uncertainty.

## 3 Results

### 3.1 Base-case results

The number of dialysis patients registered in China in 2022 was used as a parameter in the cost-effectiveness analysis of three surgical approaches for secondary hyperparathyroidism (SHPT) within the Markov model. The costs incurred by SHPT patients undergoing sPTX, tPTX, and tPTX+AT over a 10-year period after surgery were $7042.53, $9983.00, and $11435.60 respectively, with total utilities generated of 13.23 QALY, 18.76 QALY, and 18.69 QALY. Compared with sPTX, the abbreviation costs and the incremental effects of tPTX and tPTX+AT were $2940.47, $4393.07 and 5.53 QALY, 5.46 QALY respectively. The ICERs for the tPTX and tPTX+AT groups are $532.13/QALY and $805.10/QALY, respectively, both of which are below the Willingness-To-Pay (WTP) threshold of $12,700 (**Table 3**).

**Table 3:** Base case results.

Strategy	Cost ($)	Incr Cost ($)	Eff (QALY)	Incr Eff (QALY)	ICER ($/QALY)
sPTX	7042.54		13.23		
tPTX	9983.00	2940.47	18.76	5.53	532.13
tPTX+AT	11435.60	4393.07	18.69	5.46	805.10

Incr, Incremental; Eff, Effectiveness.

### 3.2 Sensitivity analysis

**Figure 2:**
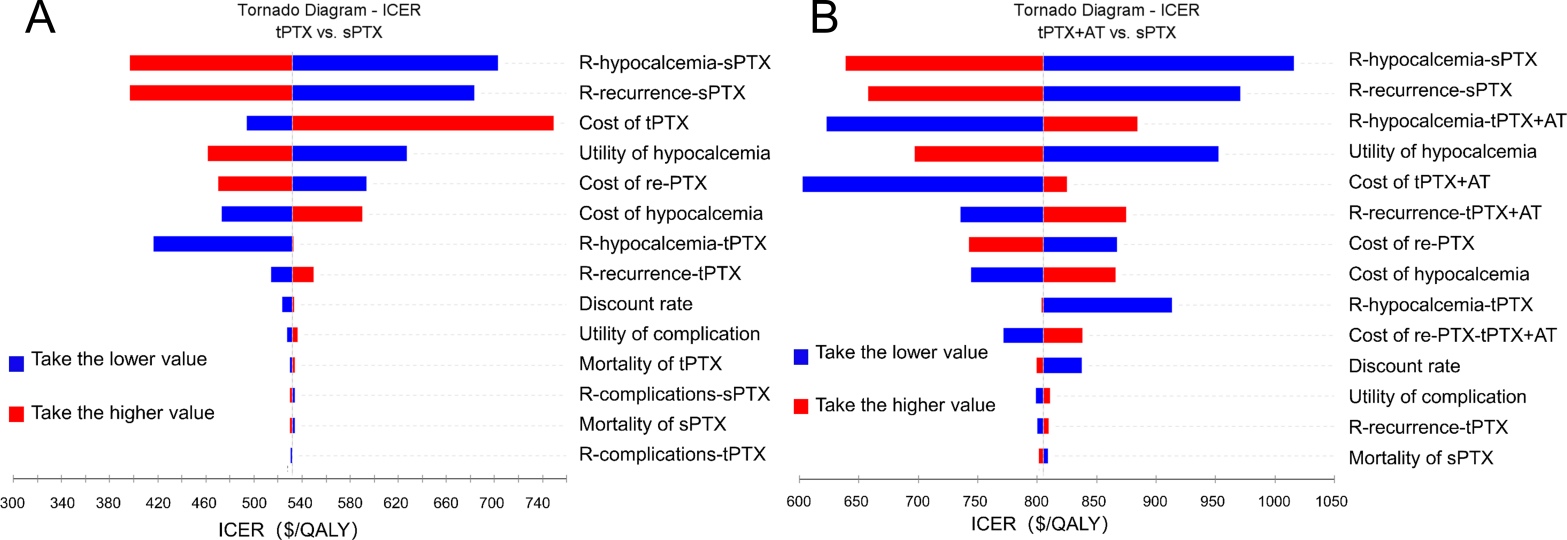
One-way sensitivity analysis. **(A)** Tornado diagram of tPTX vs sPTX; **(B)** Tornado diagram of tPTX+AT vs sPTX. R, Risk.

The tornado diagram from one-way sensitivity analysis shows that, when comparing tPTX with sPTX, the top three parameters that significantly influence ICER value are post-sPTX hypocalcemia rate, post-sPTX recurrence rate, and cost of tPTX. Moreover, the smaller values of the first two variables, the larger the ICER value becomes, whereas opposite trend is observed for the latter variable (**Figure 2A**). When comparing tPTX+AT with sPTX, the top three parameters that significantly influence ICER value are post-sPTX hypocalcemia rate, post-sPTX recurrence rate, and post-tPTX+AT hypocalcemia rate. Moreover, the smaller values of the first two variables, the larger ICER value becomes, whereas opposite trend is observed for the latter variable (**Figure 2B**). Notably, varying any parameter within a specified range over given time period does not lead to an ICER exceeding the WTP threshold nor does it reverse primary analysis results, demonstrating the robustness of the model.

**Figure 3:**
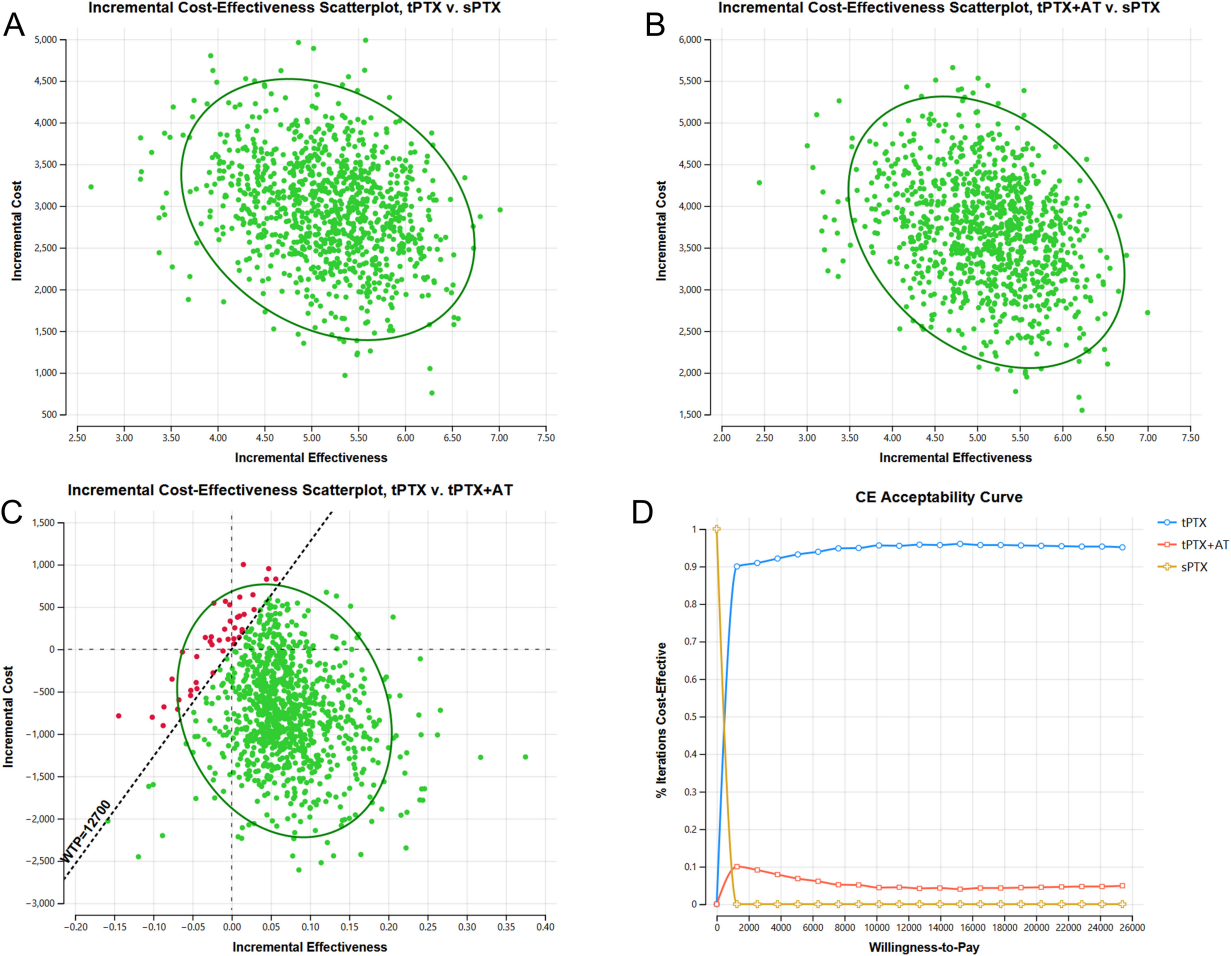
The results of probability sensitivity analysis. **(A)** Incremental cost-effectiveness scatterplot of tPTX vs sPTX; **(B)** Incremental cost-effectiveness scatterplot of tPTX+AT vs sPTX; **(C)** Incremental cost-effectiveness scatterplot of tPTX vs tPTX+AT; **(D)** Cost-effectiveness acceptability curve.

The results of probability sensitivity analysis indicate that, at the specified WTP threshold, tPTX and tPTX+AT have a 100% probability of cost-effectiveness compared with sPTX (**Figures 3A, B**). When compared with tPTX+AT, tPTX has a 95.70% probability of being cost-effective (**Figure 3C**). The cost-effectiveness acceptability curve shows that tPTX remains superior to the other two strategies, with the curve flattening as willingness to pay increases. At a specified willingness to pay of $12,700 USD, tPTX maintains cost-effectiveness in 95.7% of iterations, while tPTX+AT and sPTX maintain cost-effectiveness in 4.3% and 0% of iterations respectively (**Figure 3D**).

## 4 Discussion

Secondary hyperparathyroidism (SHPT) is a common condition among end-stage renal disease patients, resulting from metabolic disturbances. These patients often face substantial financial burdens due to the necessity of long-term or lifelong dialysis treatment or kidney transplantation. For patients with refractory SHPT, surgical treatment is the preferred option, making a selection of the most cost-effective surgical approach crucial. This study used basic data from existing clinical research to conduct an inaugural cost-effectiveness analysis of three surgical procedures for treating SHPT.

Our research findings suggest that, within the context of the Chinese healthcare system, total parathyroidectomy (tPTX) is a more cost-effective surgical treatment for patients with secondary hyperparathyroidism (SHPT) compared to the other two surgical approaches. This outcome is attributable to the relatively high risk of hypocalcemia associated with tPTX, which is offset by its lower recurrence rate, thereby reducing the likelihood of reoperation, lowering overall treatment costs, and yielding better health outcomes. These findings have significant implications for allocation of medical resources and decision-making in the management of patients with end-stage renal disease complicated by SHPT in China.

The sensitivity analysis results highlight that the post-sPTX hypocalcemia rate, post-sPTX recurrence rate, post-tPTX+AT hypocalcemia rate, and the cost of tPTX are four parameters most significantly affecting the incremental cost-effectiveness ratio (ICER). Thus, reducing postoperative hypocalcemia rates and treatment costs is crucial for enhancing cost-effectiveness. In conclusion, improving surgical techniques and perioperative management to lower the risk of postoperative hypocalcemia, or reforming the medical insurance system to reduce costs, can achieve better cost-effectiveness.

A meta-analysis incorporating 10 studies with a total of 1283 patients compared the postoperative efficacy of tPTX and tPTX+AT. The findings revealed that, in comparison to tPTX+AT, tPTX group exhibited significantly lower rates of recurrence, recurrence or persistent secondary hyperparathyroidism (SHPT), reoperation due to recurrence or persistent SHPT, and had shorter operation times. Nevertheless, tPTX group had a higher risk of hypoparathyroidism compared with tPTX+AT group, while no significant differences were observed in other aspects. Consequently, the authors suggested that tPTX might be more advantageous in treating SHPT compared to tPTX+AT (12). Additionally, a retrospective randomized controlled study of 46 patients compared the long-term and short-term outcomes of subtotal parathyroidectomy (sPTX) with tPTX+AT. The study indicated that tPTX+AT, relative to sPTX, enhances the long-term control of elevated parathyroid hormone levels and helps in preventing disease recurrence, albeit with a higher risk of long-term hypocalcemia (19). From above two studies, we can conclude that among the three surgical methods, tPTX has more advantages in surgical treatment of SHPT, which is consistent with our analysis from a cost-effectiveness perspective.

However, some researchers have presented different viewpoints. A network meta-analysis, which included 26 studies involving a total of 5063 patients, compared the three surgical methods regarding their rates of postoperative hypocalcemia (or hypoparathyroidism), recurrence, and reoperation. This study ultimately recommended tPTX+AT as the most effective and safest surgical treatment for SHPT, with the fewest adverse reactions (20), which is inconsistent with our results. This discrepancy may be due to the fact that the aforementioned studies did not account for the relationship between costs and postoperative outcomes. They overlooked the higher costs associated with reoperations due to the higher recurrence rate of tPTX+AT, which led to different conclusions compared to our study.

Although several randomized controlled studies have demonstrated that both sPTX and tPTX+AT are effective in treating SHPT, some scholars argue that CKD can stimulate the residual parathyroid glands, potentially leading to persistent or recurrent SHPT in patients. Therefore, to prevent persistence or recurrence, tPTX is considered the preferred surgical method for SHPT patients (21).

Although our study, like the aforementioned studies, found that tPTX is the optimal surgical method, other literature suggests that after kidney transplantation, stability of the internal environment reduces the stimulation of the parathyroid glands, thereby lowering the risk of SHPT. Consequently, it is recommended that patients awaiting kidney transplantation undergo tPTX+AT or sPTX to prevent hypoparathyroidism (19, 22). The KIDIGO guidelines even explicitly state that tPTX is contraindicated for patients waiting for kidney transplantation (23). Therefore, we recommend tPTX+AT for SHPT patients who are awaiting kidney transplantation.

The model designed in this study has the following limitations. First, the primary limitation of this study is that, apart from cost data, all other data in the model were derived from previously published literature rather than a purpose-built cohort study., which may lead to bias in the study results. Secondly, patients with ectopic parathyroid glands were not included in this study. which may require more extensive surgical removal. This exclusion could affect our results by influencing the costs and postoperative complications. In addition, the tPTX and tPTX+AT data included in this study were limited to cases where transcervical thymectomy was not performed during surgery. The inclusion or exclusion of thymectomy may influence the choice of the optimal surgical approach by affecting postoperative outcomes and overall costs. Finally, while the direct medical cost data were obtained from real-world on-site surveys, most of the other parameter values in the model were derived from original studies conducted internationally. Consequently, the findings of this study may have limited applicability on a global scale and are more specifically tailored to the Chinese healthcare context. Therefore, we designed one-way sensitivity analysis and probabilistic sensitivity analysis during our research to minimize the impact of these limitations. Despite these limitations, we believe that our study can provide valuable recommendations for the surgical treatment of SHPT.

## 5 Conclusion

According to our research, in Chinese healthcare system, tPTX as the first-line treatment for end-stage renal disease secondary to refractory hyperparathyroidism is considered to have the highest cost-effectiveness compared to tPTX+AT and sPTX. This conclusion is based on a comprehensive analysis of the existing data on the costs and health outcomes associated with the three different treatment strategies. However, for patients awaiting kidney transplantation, we recommend tPTX+AT. Additionally, reducing the incidence of postoperative hypocalcemia during surgical treatment is crucial for enhancing cost-effectiveness.

## Data Availability

The raw data supporting the conclusions of this article will be made available by the authors, without undue reservation.
